# Headache and pregnancy: a systematic review

**DOI:** 10.1186/s10194-017-0816-0

**Published:** 2017-10-19

**Authors:** A. Negro, Z. Delaruelle, T. A. Ivanova, S. Khan, R. Ornello, B. Raffaelli, A. Terrin, U. Reuter, D. D. Mitsikostas, on behalf of the European Headache Federation School of Advanced Studies (EHF-SAS)

**Affiliations:** 1 Department of Clinical and Molecular Medicine, Regional Referral Headache Centre, Sapienza University of Rome, Sant’Andrea Hospital, 00189 Rome, Italy; 2 Department of Neurology, Ghent University Hospital, 9000 Ghent, Belgium; 3 Institute of Professional Education, Chair of Neurology. I.M. Sechenov First Moscow State Medical University, Moscow, Russia; 4 Danish Headache Center and Department of Neurology, Rigshospitalet Glostrup, -2600 Glostrup, DK Denmark; 5 Department of Neurology, University of L’Aquila, 67100 L’Aquila, Italy; 6 Department of Neurology, Charité Universitätsmedizin Berlin, 10117 Berlin, Germany; 7 Department of Neurosciences, Headache Centre, University of Padua, 35128 Padua, Italy; 8 Neurology Department, Aeginition Hospital, National and Kapodistrian University of Athens, 11528 Athens, Greece

**Keywords:** Pregnancy, Breastfeeding, Headache, Migraine, Complications, Treatment, Adverse events

## Abstract

This systematic review summarizes the existing data on headache and pregnancy with a scope on clinical headache phenotypes, treatment of headaches in pregnancy and effects of headache medications on the child during pregnancy and breastfeeding, headache related complications, and diagnostics of headache in pregnancy. Headache during pregnancy can be both primary and secondary, and in the last case can be a symptom of a life-threatening condition. The most common secondary headaches are stroke, cerebral venous thrombosis, subarachnoid hemorrhage, pituitary tumor, choriocarcinoma, eclampsia, preeclampsia, idiopathic intracranial hypertension, and reversible cerebral vasoconstriction syndrome. Migraine is a risk factor for pregnancy complications, particularly vascular events. Data regarding other primary headache conditions are still scarce. Early diagnostics of the disease manifested by headache is important for mother and fetus life. It is especially important to identify “red flag symptoms” suggesting that headache is a symptom of a serious disease. In order to exclude a secondary headache additional studies can be necessary: electroencephalography, ultrasound of the vessels of the head and neck, brain MRI and MR angiography with contrast ophthalmoscopy and lumbar puncture. During pregnancy and breastfeeding the preferred therapeutic strategy for the treatment of primary headaches should always be a non-pharmacological one. Treatment should not be postponed as an undermanaged headache can lead to stress, sleep deprivation, depression and poor nutritional intake that in turn can have negative consequences for both mother and baby. Therefore, if non-pharmacological interventions seem inadequate, a well-considered choice should be made concerning the use of medication, taking into account all the benefits and possible risks.

## Introduction

Headache is the most frequent referral for neurologic consultation in the outpatient setting. The last release of data at 2013 from the Global Burden of Disease (GBD) - described now as “the most comprehensive worldwide observational epidemiological study to date” [1] - established headache disorders collectively as the seventh highest cause of years lived with disability (ylds) [2].

In front of a patient complaining about headache, the first purpose is to distinguish a primary headache (when pain *is the disease*) from a secondary headache (when pain *is a symptom of another disease*). More strictly, this is the main concern with a pregnant woman suffering from this symptom. Three scenarios are possible [3, 4]:

1. She suffers from a primary headache and now she presents with her usual headache;
2. She does not suffer from a primary headache and she presents with her first severe headache during pregnancy;
3. She suffers from a primary headache, but now pain is different in quality, intensity or associated symptoms.

In the second and third scenarios, headache must be considered as a symptom of an underlying disease until an appropriate diagnostic evaluation has been performed.

This systematic review is a summary of existing data on headache and pregnancy with a focus on clinical headache phenotypes, treatment of headaches in pregnancy and effects of headache medication on the child during pregnancy and breastfeeding, headache-related complications, and diagnostics of headache in pregnancy.

## Methods of review

Two independent reviewers conducted an independent search on pubmed using the search terms “pregnancy” and “headache” OR “migraine”, each combined with “complications” OR “treatment” OR “management”. This search was carried out on June 15th, 2017. We included articles from the past 20 years. The initial screening was conducted based on eligibility of titles and abstracts. Original works, randomized, placebo- or comparator-controlled trials, published in full, were primarily selected for the review. Other references quoted include: systematic reviews, open label studies, retrospective studies, population-based studies, guidelines, manufacturers product monographs and letters to the editor. Discrepancies between reviewers were resolved by discussion.

## Clinical headache phenotypes and observational studies in pregnancy

### Primary headaches

In most cases headache is a primary disorder, including migraine and tension-type headache (TTH) as the more frequent conditions that affect women asking medical consultation. Several observational studies have been conducted to evaluate the course of primary headaches during pregnancy (Table 1). During pregnancy, primary headaches also showed a tendency to change in pattern from migraine without aura (MO) to migraine with aura (MA) and vice versa or from MO to TTH and vice versa: in an Italian study 9% of TTH patients developed MO during gestation, while 10% did the opposite [5]. Up-to-date, TTH is not correlated with any adverse pregnancy outcomes, even if sample size of the available studies are too small to achieve definitive conclusions [4].

**Table 1:** Primary headaches course during pregnancy

Author	Study design	Sample size	Improvement or remission (%)	Unchanged (%)	Worsening (%)	Extra data
Migraine without aura						
Granella et al. [8]	R	571	67.3	29.2	3.5	Full sample size: 1300 women; 943 had had pregnancies; 571 women with migraine before first pregnancy
Scharff et al. [9]	P	19	56.7	36.6	6.7	Full sample size: 30; 11/30 with headache onset during pregnancy
Maggioni et al. [5]	R	81	89.5	7.7	2.5	Full sample size: 430 women, interviewed 3 days after delivery; among them, 81 MO, 12 MA, 33 TTH
Marcus et al. [10]	P	49	40.8	51	8.2	16 M, 16 TTH, 15 M + TTH. Headache recorded daily during pregnancy and 3 months post-partum
Granella et al. [11]	R	200	76.8	22.2	1	100 MA and 200 MO as controls
Mattsson [12]	R	728	81.4	17.6	1	Full sample size: 728; full information available for 102 women
Sances et al. [13]	P	47	87.2	12.8	0	Full sample size 49: 2 MA, 47 MO
Kelman [14]	R	504	38.2	27.8	34	Greater improvement in MO patients rather than MA patients
Ertresvåg et al. [15]	P	410	65.9	19.8	14.4	Full sample size: 1361 women. 410 with M.
Melhado et al. [16]	P	737	65	26.1	8.9	Full sample size: 1101 women. 737 with M. Data partially derived from graphics
Summary		3346	66.9	25.8	8	
Migraine with aura						
Maggioni et al. [5]	R	12	83.4	16.6	0	430 women 3 days after delivery; among them, 81 MO, 12 MA, 33 TTH
Granella et al. [11]	R	100	43.6	48.7	7.7	100 MA and 200 MO as controls
Mattsson [12]	R	728	78.3	4.3	17.4	Full sample size: 728; full information available for 23 women
Summary		840	68.4	23.2	8.4	
Tension-type Headache						
Maggioni et al. [5]	R	33	82,1	17,9	0	Full sample size: 430 women, interviewed 3 days after delivery; among them, 81 MO, 12 MA, 33 TTH
Melhado et al. [16]	P	112	N/A (≈ 60)	N/A (≈ 35)	N/A (≈ 5)	Full sample size: 1101 women. 112 with TTH. Data derived from graphics
Summary		145	–	–	–	
Cluster Headache						
Van Vliet et al. [31]	R	53	69,9	20,7	9,4	Full sample size: 196 CH; 53 had their first attack before the first pregnancy. 23% of episodic CH patients reported that an “expected” cluster period did not occur during pregnancy. Here improvement includes 8 patients who had a cluster period within 1 month after delivery.

*M*, migraine; *MO*, migraine without aura; *MA*, migraine with aura; *TTH*, tension type headache; *CH*, cluster headache

### Migraine

On the wake of the first pioneering articles [6, 7], following retrospective and prospective studies dealing with migraine and pregnancy published in the last twenty years show similar results. About one half to three fourths of female migraineurs experience a marked improvement in migraine during pregnancy with a significant reduction in frequency and intensity of their attacks, if not a complete resolution (Table 1) [8–18]. The remaining attacks show a progressive reduction in the mean pain intensity and duration as pregnancy proceeds [13, 17]. As a consequence, the 1-year headache prevalence of migraine and non-migrainous headache is lower among nulliparous pregnant women than in non-pregnant women [19]. Maggioni et al. Reported an absolute improvement during the first trimester, with a further reduction during the second and third ones [5], a data that has been confirmed by more recent studies [9, 13]. Differently, the Head-HUNT study found a marked reduction in headache burden only in the third trimester [19]. About 50% of the pluripara mothers present a persistent worsening of their headache with following gestations [5]. This is in line with the evidence that multiparous subjects more likely experience worsening of headache [9]. Other studies showed no significant differences between primi- and multiparous pregnant women as regards the course of headaches during gestation among migraineurs [17, 19], neither confirming the trend of further improvement after the first trimester [10]. A large Italian study found that the percentage of remissions during pregnancy was significantly higher in the subgroup of patients whose migraine started at menarche and in those suffering from menstrual migraine [8], even if this last data has not been confirmed by following studies [10, 13, 14].

Migraine without aura (MO) can start during pregnancy in 1 up to 10% of pregnant women [5, 15–17, 20], with some retrospective data rising up to 16.7% [13]; this is classically considered a first trimester phenomenon [5–7, 20]. In other cases, migraine can worsen during pregnancy, especially in the first trimester: this is reported in 8% of cases (Table 1) [5, 8–16]. Except for a few works concerning headache frequency [5, 13, 15], most articles analyse headache modifications during pregnancy without distinguishing between frequency and intensity of the attacks. A mean of 25% of MO patients will continue to have attacks during pregnancy, with hyperemesis, pathological pregnancy course and pre-gestation menstrual-related migraine being linked with this lack of improvement [13, 21]. Up-to-date, scientific literature lacks of large and rigorous studies aimed at understanding factors possibly associated with the absence of a clinical improvement during pregnancy [22, 23].

The relationship between estrogens fluctuations and MA has been the focus of fewer studies. MA starts or worsens during pregnancy more frequently than MO does: onset during gestation is reported in 10.7 up to 14% of cases [5, 11], worsening covers 8.4% of MA women (Table 1), with “no change” in pain pattern representing the most frequent evolution during gestation. Nearly half patients with MA will continue to have attacks [11]. This trend to recede in a lower number of cases than MO has been transversely confirmed [5, 13, 14], with rare exceptions [16]. It could be due to increased endothelial reactivity in MA patients compared with MO ones [21]. MA can develop new aura symptoms during gestation [24, 25], as pregnancy may trigger attacks of aura without headache as well [26]. In less frequent cases, hemiplegic migraine makes the differential diagnosis very difficult, especially in the third trimester [27, 28]. Therefore, we can easily understand why transient neurologic symptoms during pregnancy are more common among pregnant women with migrainous headache than in those without headache or with non-migrainous headache [15].

Postpartum headache occurs in about 30–40% of all women, not only migraineurs [6, 9, 13]. Most of the attacks develop during the first week after delivery, with apparent sparing of the day of birth. During puerperium mean headache intensity, pain duration and analgesic therapies increase, as confirmed by a large prospective trial [17]. On the other side, the MIGRA study showed a decline in attacks frequency starting five weeks after delivery [17]. None of the migraineurs experiencing a complete pain remission during the first or the second trimester should experience a recurrence of migraine attacks before delivery [13], even if a study reported an increase in headache burden already in the four weeks before birth in multiparous women, defining a U-shaped curve to describe migraine evolution during pregnancy [9]. A large multicentre study set at 3.7% the amount of women experiencing headache within 72 h after delivery, identifying headache during pregnancy and regional anaesthesia injections as risk factors [28]. Migraine usually returns quickly after delivery, probably triggered by the abrupt fall in the level of estrogens, by a postpartum depression or because of the new parental role and all that it implies (sleep deprivation, anxiety, worry and psychological adaptation). Pre-pregnancy headache pattern restores within 1 month from delivery in 55% of patients and only breast-feeding and age > 30 years have been reported to retard headache recurrence [13]. Migraine recurred within the first post-partum month in 100% of women who bottle-fed and in only 43.2% of those who breastfed [13]. However, other studies found no significant association between headache improvement from the second trimester to the postpartum and breastfeeding [10, 17].

At present only one study dealt with the headache attacks during the course of in vitro fertilization and embryo-transfer treatments [29]. The prevalence of headache attacks is higher at the first stage of the procedure (with gnrh analogue administration) and at the end of the treatment protocol in cases there is no conception, in both situations because of a decline in blood levels of estrogens.

### Tension-type headache

TTH represents 26% of headaches in pregnancy [30]. TTH would be expected to improve during gestation as female hormones modulate serotonin and endorphins, which are involved in TTH pathophysiology [4]. Actually, 17.9% of TTH patients reported no change in the headache burden during pregnancy (Table 1), with worsening in 5% of cases and improvement in a quarter of women [3, 30]. A study found significantly higher remission and improvement rates than in MO (Table 1) [5]. Whatever if women with TTH showed a great or a modest improvement, this is usually reported as marked as for the migraineurs [10, 16]. On the contrary, TTH rarely worsens during gestation [16] and, according to some Authors, it never does [5].

### Cluster headache

Cluster headache (CH) is a relatively rare primary headache, severe in intensity, stabbing in quality, highly debilitating, associated with autonomic symptoms and affecting men more frequently than women. Scientific literature lacks of large prospective studies about the effect of pregnancy on CH, as it is seen in less than 0.3% of pregnancies [30]. Despite the rare cases in which the first attacks occur during the first pregnancy, almost a quarter of pregnant women report that an expected cluster period does not develop during gestation while it may start soon after delivery [31]. Otherwise, CH attacks do not change in intensity and frequency in the majority of cases. As a consequence, women who have their first attack before their first gestation usually have fewer children than those who already were mother at the time of clinical onset; this is probably due to the prospective of the treatment limitations in case of CH during pregnancy [31].

### Secondary headaches

Pregnancy is a risk factor for a secondary headache disorder. Hypercoagulability, hormonal changes and anaesthesia for labour are just some of the multiple factors contributing to the high incidence of secondary headaches during pregnancy.

A recent study by Robbins et al. Found 35% of secondary headaches among 140 pregnant women presenting with acute headache: hypertensive disorders of pregnancy covered 51% of these cases (about 18% of total), with preeclampsia as the major cause, followed by reversible posterior leukoencephalopathy syndrome (PRES, eclampsia), reversible cerebral vasoconstriction syndrome (RCVS) and acute arterial hypertension [32]. These data place between two previous studies that reported percentages of secondary headaches ranging from 14.3% to 52.6% [16, 33]. In particular, among patients with a primary headache history, longer attack duration is the most common feature suggesting a secondary headache, reaching the statistical significance [32] or just approaching it [33].

The authors show how lack of headache history, elevated blood pressure and abnormalities at neurologic examination are the main red flags for a secondary origin of an acute headache during pregnancy [32]. In the second trimester, a new onset of headache may signal the presence of a pseudotumor cerebri [34], while in case of a severe postural headache a spontaneous intracranial hypotension must be ruled out [35]. In front of the well-known red flags (Table 2) brain MRI or CT scan are often required [30, 36]. Use of contrast agents such as gadolinium is not recommended, given the lack of data regarding safety to the fetus and its ability to cross the placenta and remain in the amniotic fluid [30].

**Table 2:** Red Flags for headache in pregnancy

1.	Headache that peaks in severity in less than five minutes
2.	New headache type versus a worsening of a previous headache
3.	Change in previously stable headache pattern
4.	Headache that changes with posture (e.g. Standing up)
5.	Headache awakening the pregnant
6.	Headache precipitated by physical activity or Valsalva manoeuvre (e.g. Coughing, laughing, straining)
7.	Thrombophilia
8.	Neurological symptoms or signs
9.	Trauma
10.	Fever
11.	Seizures
12.	History of malignancy
13.	History of HIV or active infections
14.	History of pituitary disorders
15.	Elevated blood pressure
16.	Recent travel at risk of infective disease

Modified from Mitsikostas et al. 2015 [38] (European Headache Federation consensus on technical investigation for primary headache disorders)

Iodinated contrast should be avoided as well as it may suppress fetal thyroid function [37].

Recently the European Headache Federation (EHF) published a consensus statement on technical investigation for primary headache disorders [38].

Secondary headache features may not differ from those of primary headaches; furthermore, migraine is as an independent risk factor for the development of secondary headaches (e.g., the risk of gestational hypertension increased by 1.42-fold with an OR of 2.3 (CI 2.1–2.5) [39], so that recognizing these conditions in pregnant women may be a true diagnostic challenge.

Cerebral venous thrombosis (CVT), pre-eclampsia, haemorrhagic or ischemic stroke, subarachnoid haemorrhage (SAH), RCVS, PRES, idiopathic intracranial hypertension (IIH) or pituitary apoplexy must be ruled out as soon as possible (Table 3) [3, 37].

**Table 3:** Main causes of secondary headache in pregnant women

Secondary headaches during pregnancy	
Arterial dissection	Intracranial hypotension
Arteriovenous malformation	Ischemic stroke
Brain tumors	Meningitis/encephalitis
Cerebral venous thrombosis (CVT)	Pituitary adenoma
Choriocarcinoma	Pituitary apoplexy
Cranial neuralgias	Pituitary meningioma
Dehydration	Reversible posterior leukoencephalopathy syndrome (PRES)
Eclampsia and pre-eclampsia	Reversible vasoconstriction syndrome (RCVS)
Head trauma	Sinusitis
Idiopathic intracranial hypertension (IIH)	Subarachnoid haemorrhage (SAH)
Intracranial haemorrhage (ICH)	Vasculitis

### Cerebral venous thrombosis

Headache caused by CVT has no specific characteristics: it is most often diffuse, progressive and severe, but can be unilateral and sudden (even thunderclap), or mild, and sometimes is migraine-like [40]. Headache is present in 80–90% of cases of CVT and it is often associated with focal signs (neurological deficits or seizures) and/or signs of intracranial hypertension (nausea and papilledema), subacute encephalopathy or cavernous sinus syndrome, carrying a mortality rate of up to 30% [30].

### Pre-eclampsia and eclampsia

Pre-eclampsia occurs in 5% of pregnancies [30]: a progressive bilateral (temporal, frontal, occipital or diffuse) pulsating headache in a woman who is pregnant or in the puerperium (up to 4 weeks postpartum), often aggravated by physical activity, failing to respond to the over-the-counter remedies, may be the herald symptom of this condition, which can associate with visual changes similar to the typical visual aura. It must resolve within a week after blood pressure adjustment [4, 41]. According to the International Classification of Headache Disorders (ICHD-3beta) headache should have at least two of the following three characteristics: a) bilateral location, b) pulsating quality, and c) aggravated by physical activity [42].

### Ischaemic stroke

Headache accompanies ischaemic stroke especially within the posterior circulation, in up to one-third of cases and is usually overshadowed by focal signs and/or alterations of consciousness, which in most cases allows easy differentiation from the primary headaches. The risk of ischaemic stroke in migraineurs was evaluated using the United States (US) Nationwide Inpatient Sample from the Healthcare Cost and Utilization Project of the Agency for Healthcare Research and Quality and found to be elevated [39].

Headache has a self-limited course, and is very rarely the presenting or a prominent feature of ischaemic stroke [43]. It is usually of moderate intensity, and has no specific characteristics. It can be bilateral or unilateral ipsilateral to the stroke. Rarely, an acute ischaemic stroke can present with an isolated sudden (even thunderclap) headache [44].

The diagnosis of headache and its causal link with ischaemic stroke is easy because the headache presents both acutely and with neurological signs and because it often remits rapidly.

### Subarachnoid haemorrhage

In SAH headache is usually the prominent symptom. The pain is typically severe and sudden, peaking in seconds (thunderclap headache) or minutes, often followed by vomiting and loss of consciousness [45]. SAH is a serious condition with mortality rate of 40–50% and with 10–20% of patients dying before arriving at hospital. The abrupt onset is the key feature and can help to distinguish from primary headaches with thunderclap features (e.g., associated with exercise or sexual activity). SAH presents a 20-fold increased risk in the puerperium and it gives a thunderclap headache [30].

### Arterial dissection

Arterial dissection is a rare complication of pregnancy and puerperium. There have been reports of cervical carotid, vertebral and intracranial artery dissection in association with preeclampsia [46]. Headache is the most frequent inaugural symptom, described as severe, unilateral (ipsilateral to the dissected vessel), with a sudden (even thunderclap) onset. Pain is persistent for days and can remain isolated or be a warning symptom preceding ischaemic infarcts.

### Reversible cerebral vasoconstriction syndrome

Headache caused by RCVS syndrome is severe and diffuse and typically of the thunderclap type, recurring over 1–2 weeks, often triggered by sexual activity, exertion, Valsalva manoeuvres and/or emotion [47]. Headache is often the only symptom of RCVS, but the condition can be associated with fluctuating focal neurological deficits and sometimes seizures.

RCVS is commonly associated with the post-partum period, usually within a week after delivery: its severe thunderclap headache usually relapses within a few days, resolving by approximately twelve weeks after clinical onset [21, 37]. The typical differential diagnosis is cerebral vasculitis, which needs to be ruled out due to the course of the disease and different treatment options.

### Posterior reversible encephalopathy syndrome

PRES is a neuro-radiological clinical entity characterized by insidious onset of headache, impaired consciousness, visual changes or blindness, seizures, nausea, and vomiting, and focal neurological signs. In nearly 2/3 of patients with PRES, headache is the most common symptom and is usually described as occipital and bilateral and dull in nature [48]. Symptoms develop without prodrome, and progress over 12–48 h.

PRES is often associated with hypertensive encephalopathy, preeclampsia, eclampsia, RCVS, renal failure, immunosuppressive therapy or chemotherapy. PRES is more common in women and the development of this condition after delivery is unusual. The condition is usually reversible when early diagnosis is established and appropriate treatment is started without delay; symptoms generally resolve within a period of days or weeks while recovery of the MRI abnormalities takes longer [49].

### Idiopathic intracranial hypertension

Usually during the first trimester, obese women can suffer from a progressive, daily headache, aggravated by Valsalva and position change, associated with papilledema and severe visual deficits, together with tinnitus or sixth-nerve palsies, defining the clinical pattern of IIH [30, 37, 50]. The headache is frequently described as frontal, retro-orbital, ‘pressure like’ or explosive; migraine-like headache may also occur.

### Pituitary apoplexy

Pituitary apoplexy is a rare cause of sudden and severe headache during pregnancy [51]. The sudden rise of a severe headache, with nausea, vomiting, ophtalmoplegia, altered consciousness and accompanied from onset or later by visual symptoms and/or hypopituitarism must raise the clinical suspicion of a pituitary apoplexy [4, 50]. The rare clinical syndrome of pituitary apoplexy is an acute, life-threatening condition. It is important to distinguish from the other causes of thunderclap headache [52]. Most cases occur as the first presentation of rapid enlargement of non-functioning pituitary macroadenomas as a result of haemorrhage and/or infarction.

## Treatment of headaches in pregnancy and breastfeeding women

During pregnancy, inadvertent exposure to teratogenic agents can lead to irreversible fetal malformations [53, 54]. Unfortunately, most patients are not aware of possible teratogenic risks of used medications and their safety profiles during pregnancy [55].

During pregnancy and breastfeeding the preferred therapeutic strategy should always be a non-pharmacological one. Nevertheless, an undermanaged headache can lead to stress, sleep deprivation, depression and poor nutritional intake which in turn can have negative consequences for mother and baby. Therefore, if non-pharmacological interventions seem inadequate, a well-considered choice should be made concerning the use of medication, taking into account all the benefits and possible risks (Tables 4 and 5). A basic rule should be to aim for the lowest effective dose and the shortest duration of treatment.

**Table 4:** Summarizing table on treatment of headache in pregnant women

Medication	Adverse effects	Concerns	Comments
Paracetamol	–	Possible increased risk for asthma, ADHD	Preferred acute treatment
Nsaids (non-selective): ibuprofen, naproxen, diclofenac, indomethacin	- TR1: miscarriage - TR3: premature closure ductus arteriosus, impaired renal function, cerebral palsy, intraventricular haemorrhage	TR1: possible associated CM	- can be used safely during TR2 - avoid in TR3 - selective COX-inhibitors contra-indicated
Triptans: sumatriptan, zolmitriptan, eletriptan, rizatriptan	No major congenital defects	TR1: possible link with behavioral problems	Appropriate if benefit outweighs risk
Aspirin (ASA)	> 100 mg/d or TR3: premature closure of ductus arteriosus, oligohydramnios, neonatal bleeding	–	- < 100 mg/day seems safe - caution in TR1 and TR2 - avoid in TR3
Caffeine	–	Moderate to high daily doses: possible association with miscarriage, low birth weight, preterm delivery	–
Combined preparations: paracetamol, aspirin and caffeine	–	–	Not recommended
High flow oxygen	–	–	Preferred acute treatment in CH
Lidocaine	–	–	- second line acute treatment in CH - intranasal formulation preferred
Corticosteroids: prednisone, prednisolone	–	Possible early lung maturation	- avoid during first semester - low doses recommended - reserved for CH or status migrainosus
Weak opioids: tramadol, codeine	- MOH - withdrawal symptoms and respiratory depression in the newborn	–	- not considered first line treatment in primary headaches - caution in TR1 and TR2 - avoid in TR3
Ergots/Ergots Alkaloids	- uterotonic and vasoconstrictive effect - fetal distress - CM	–	Avoid in any trimester
Β-blockers: metoprolol, propranolol	Neonatal bradycardia, hypotension, hypoglycaemia when exposed in TR3	- intrauterine growth retardation - preterm birth - respiratory distress	- first line migraine prophylaxis - if possible tapper off TR3 - monitor newborn exposed in TR3
ACE- I, ARB	CM	–	Avoid in any trimester
Verapamil	–	–	First line CH profylaxis
TCA	–	- possible CM (not confirmed) - withdrawal symptoms in the newborn	- second line migraine prophyaxis when β-blocker ineffective/contra-indicated - amytriptiline preferred
Venlafaxine	CM	–	Should be avoided
Duloxetine	–	–	No reported AE
Valproate	Neural tube defects, cardiac defects, urinary tract defects, cleft palate, lower IQ scores	–	Avoid in any trimester
Topiramate	Cleft lip/palate, low birth weight	–	Avoid in any trimester
Gabapentin	–	Osteological deformities	Limited data
Lamotrigine	No major congenital defects	Increased occurrence of autism/dyspraxia	Safest antiepileptic drug
Magnesium	- high dose I.V.: bone abnormalities - possible transient neurological symptoms and hypotonia after delivery	Possible bone abnormalities in lower dosage or when taken orally	- appropriate in any trimester; caution directly before delivery - chronic use of oral magnesium: controversial
Coenzyme Q10	–	–	No reported AE
Feverfew, butterbur, high dosed riboflavine	–	Possible CM	Not recommended
Flunarizine	–	–	Not recommended (no data available)
Lithium	- congenital cardiac malformations and cardiac arrhythmias - anomalies of the CNS and endocrine system - polyhydramnios - stillbirth	–	Not recommended but can be considered in uncontrolled CH refractory to Verapamil
Botulinum toxin A	–	–	No reported AE when injected correctly
Nerve blocks	–	–	- no reported AE when injected correctly - preferred agent: lidocaine

Adverse effects are the known proven side effects. Concerns cover issues that are presumed based on limited data but for which the causal relationship is not clear

*TR1*, first trimester; *TR2*, second trimester; *TR3*, third trimestes; *AE*, adverse effects; *ADHD*, attention-deficit/hyperactivity disorder; *CM*, congenital malformation; *CH*: cluster headache, *TCA*, tricyclic antidepressants; *ACE-I*, ACE-inhibitor; *ARB*, angiotensin-receptor blocker; *I.V*., intravenously

**Table 5:** Summarizing table on treatment of headache in breatsfeeding women

Medication	Adverse effects	Comments
Paracetamol	–	Preferred acute treatment
Nsaids (Non-selective): Ibuprofen, naproxen, indomethacin	Aggravation of jaundice	Ibuprofen preferred
Triptans	–	- sumatriptan: no need to ‘pump and dump’ - less evidence on the other triptans: avoid nursing for 24 h after use of triptan as extra safety measurement
Aspirin (ASA)	Reye’s syndrome	Not recommended
Caffeine	–	Moderate dosage
High flow oxygen	–	Preferred acute treatment in CH
Lidocaine	–	- second line acute treatment in CH - intranasal formulation preferred
Corticosteroids: prednisone, prednisolone	Prolonged high-dosed therapy: infant growth and development can be affected	Intravenously: delay breastfeeding for 2-8 h
Weak opioids: tramadol, codeine	Sedation and respiratory depression in the infant	Not considered first line treatment in primary headaches
Ergots/Ergots Alkaloids	- vomiting, diarrhea, convulsions - decrease of prolactine in the mother	Avoid in any trimester
Β-blockers: metoprolol, propranolol	- hypotension, bradycardia - weakness	- metoprolol preferred - avoid in children with astma
ACE-I, ARB	Impact on kidney development	Probably compatible (limited data)
Verapamil	–	First line CH profylaxis
TCA	–	No reported AE
Venlafaxine	–	No reported AE
Duloxetine	–	No reported AE
Valproate	Interfere with liver and platelet function	Avoid in women of child-bearing age
Topiramate	- sedation, irritability - poor suckling, diarrhea	–
Gabapentin	–	No reported AE
Lamotrigine	–	No reported AE
Magnesium, Riboflavine	–	No reported AE
Flunarizine	–	Not recommended: no data available
Lithium	Renal toxicity	Not recommended, but can be considered in uncontrolled CH, refractory to Verapamil
Botox	–	No reported AE when injected correctly
Nerve blocks	–	No reported AE when injected correctly

Adverse effects are the known proven side effects. Concerns cover issues that are presumed based on limited data but for which the causal relationship is not clear

*TR1*, first trimester; *TR2*, second trimester; *TR3*, third trimestes; *AE*, adverse effects; *ADHD*, attention-deficit/hyperactivity disorder; *CM*, congenital malformation; *CH*: cluster headache, *TCA*, tricyclic antidepressants; *ACE-I*, ACE-inhibitor; *ARB*, angiotensin-receptor blocker; *I.V*., intravenously

Medication is considered safe during breastfeeding if the relative infant dose is <10% [36, 56]. The risk of adverse events could be minimized by taking medication directly after breastfeeding and discarding all milk for at least 4 h [18].

### Non-pharmacological treatment

Triggers like sleep deprivation, skipping meals and emotional stress should be avoided. A balanced lifestyle with attention for physical activity and regular eating and sleeping habits is recommended [37, 57–62]. Acupuncture and behavioral therapies like biofeeback and yoga can be helpfull [37, 58, 62]. In particular for women with CH screening for sleep apnea is usefull, since prevelance seems higher in cluster patients and in pregnancy [63]. Treatment with a dental device or continuous positive airway pressure can be proposed [57].

### Symptomatic treatment

#### Paracetamol/acetaminophen

Paracetamol is considered the safest option to treat acute pain during pregnancy and breastfeeding [37, 50, 54, 59–62, 64, 65]. Despite this historical reputation, new data suggesting a possible relationship between prenatal exposure to paracetamol and an increased risk of asthma and attention-deficit/hyperactivity disorder (ADHD) in the child raise some concern [37, 60, 62]. Patients should be educated about the difference between paracetamol and combinated drugs containing paracetamol and other substances, like codeine or caffeine.

#### Aspirin

The use of acetylsalicylic acid (ASA) in low doses (< 100 mg/day) does not seem to induce any associated maternal or neonatal complications. However, higher doses should be avoided as well as its use in the third trimester, since there might be a link with premature closure of the ductus arteriosus and oligohydramnios [20, 50, 53, 54, 60]. Due to its effect on platelet function, ASA can also increase the risk of neonatal bleeding [18].

Breastfeeding women should be discouraged to use ASA due to a potential toxicity. It is associated with Reye’s syndrome. A potential adverse effect on platelet function in the infant is suspected, but remains unclear [37, 59, 61, 64].

#### Caffeine

In animal studies a teratogenic effect of high-dosed caffeine was demonstrated. On the other hand, caffeine-containing beverages are consumed very commonly during pregnancy, without any reported adverse effect. In general the use of caffeine in low doses is assumed to be safe. Moderate-to-high daily doses are more controversial since they might be associated with miscarriage, low birth weight and preterm delivery [60, 62, 66]. Combined preparations containing paracetamol, aspirin and caffeine should be avoided [50].

Moderate intake of caffeine seems safe for mother and child when breastfeeding [64].

#### Non-steroidal anti-inflammatory drugs

Attention should be paid to the timing of the pregnancy and the type of non-steroidal anti-inflammatory drugs (nsaids) used. Non-selective COX-inhibitors like ibuprofen, naproxen and diclofenac can be a relative safe choice in the second trimester. Nsaids are not recommended in the third trimester since there is an increased risk of complications like premature closure of the ductus arteriosus, impaired renal function, cerebral palsy and neonatal intraventricular haemorrhage [50, 61, 62, 66]. More recent data suggest to avoid nsaids during the first trimester as well. An increased risk of miscarriage is suspected when used close to conception based on available reports and seems plausible regarding the pharmacological properties of this drug. Different studies covering over 20.000 pregnancies reported on the association between congenital malformation and prenatal nsaids exposure in the first trimester. Some population-based studies confirm the association, but others do not [37, 60, 61]. Selective COX2-inhibitors are contraindicated in pregnancy based on the few prenatal data available [60].

Naproxen, indomethacin and ibuprofen are compatible with breastfeeding, prefering ibuprofen because of its short elimination half-life and low excretion in human milk. In newborns with jaundice nsaids exposure can exacerbate the condition [37, 60, 61, 64].

#### Triptans

Considerable data is available on the use of sumatriptan in pregnancy. A few large pregnancy registries covering more than 3000 pregnancies, retrospectively analyse the use of other triptans, in particular rizatriptan, zolmitriptan and eletriptan [37, 56]. Due to its small molecular weight, sumatriptan can pass through the placenta [67]. However, the transfer is slow and passive, so that only about 15% of maternal dose reach the fetus after 4 h [68]. Ephross et al. Reported the last data from the sumatriptan and naratriptan pregnancy registries [69]. Until 2012, the registry included 680 exposed women, giving birth to 689 fetuses. 90.9% of them were exposed to sumatriptan. The overall risk of major birth defects under sumatriptan exposure was 4.2%. The most common birth defects were ventricular septal defect (*n* = 4), pyloric stenosis (*n* = 3) and chromosomal abnormalities (*n* = 5). The authors concluded that sumatriptan does not lead to teratogenity, as risk rates for major birth defects were similar to general population (3–5%). Only one major birth defect, i.e. Ventricular septal defect, occurred under naratriptan exposure during first trimester in a fetus who was also exposed to sumatriptan. The number of newborns exposed to naratriptan was too low to allow accurate interpretation [69]. In the rizatriptan registry 4 major birth malformations occurred in 56 pregnancies (7.1%). Also in this case data are currently too scarce to draw any conclusion [56, 70]. Observational studies conducted in Denmark, Sweden and Norway reported no increased risk for fetal malformations under triptan use [67, 71–73]. However, children exposed to triptans in utero might have a higher risk of developing externalizing behaviors [74]. Exposure to triptans in late pregnancy is associated with an increased risk of atonic uterus and postpartum haemorrhage [37, 56, 60, 61, 69, 72, 73, 75, 76]. In their meta-analysis, Marchenko et al. Concluded that triptans do not lead to increased rates of major congenital malformations [76]. The rates of spontaneous abortions were elevated when compared to healthy controls (OR 3.54), but not with untreated migraineurs [76]. Entries in pregnancy registries are voluntary and therefore not systematic. Most registers and observational studies do not include sufficient data about how often triptans were taken, exposure to concomitant medications or severity of illness as a possible confounders [76–78]. Some concern exists on a potential increased risk of behavioral problems like attention deficit and aggression disorders after prenatal exposure to triptans, in particular in the first trimester [74].

The use of sumatriptan is compatible with breastfeeding without the need to “pump and dump”. The infant exposure is very low corresponding to 0.5% of maternal dose and no adverse events on the nursing infant were reported [77]. Theoretically, eletriptan can be considered even safer as the dose in breast milk is only 0.002% after 24 h [36]. Clear controlled evidence on the other triptans is lacking. They are considered probably compatible with breastfeeding [37, 57, 59, 61, 62, 64, 79]. As an extra safety measure it can be advised to avoid breastfeeding for 24 h after their use [59].

#### Oxygen

In pregnant and breastfeeding women with CH, high flow oxygen administered via a non-rebreathing mask is the preferred treatment. It seems a safe option without proven adverse effect on the child or the mother [50, 57, 79, 80].

#### Lidocaine

The use of lidocaine is a considerable option for pregnant women with CH, when treatment with oxygen is insufficient [50, 57, 80]. The intranasal formulation is preferred since it is presumed to have a better safety profile than the systemic formulations [80].

Lidocaine is compatible with breastfeeding in any formulation [57, 64, 79, 80].

#### Corticosteroids

There is some concern about early lung maturation and a slightly increased risk for cleft palate, but in disabilitating CH and status migrainosus prednisone and prednisolone remain a reasonable alternative [57, 60, 65, 80]. Therefore, they should be avoided during first trimester and the dose should be kept as low as possible [80].

Oral prednisone and prednisolone are compatible with breastfeeding as only about 1–2% of the mother dose transfers to the fetus [64]. Prolonged high-dosed therapy should be avoided since infant growth and development could be affected [57, 64]. When administered intravenously, breastfeeding should be delayed untill 2 to 8 h after administration [80].

#### Opioids

Weak opioids like tramadol and codeine can be considered when non-opioid medication brings no relief [37, 61, 66, 81]. Codeine was initially supposed to increase the risk for cleft palate and inguinal hernia but large observational studies could not confirm it [81]. A slightly higher risk for cardiac defects or spina bifida has been described after opioid exposure in first trimester [61]. Prolonged use of such drugs should be clearly discouraged because of the risk of medication overuse headache (MOH) for the mother and dependency with withdrawal syndrome in the newborn [37, 62]. Stronger formulations should be used with caution and opioid use is discouraged during third trimester, since narcotics cross the placenta and can cause fetal bradycardia, respiratory depression and birth defects [61, 62].

Sporadic use of weak opioids is compatible with breastfeeding. When repeated dosing or highly dosed opioids are needed, there is a risk of sedation, respiratory depression and constipation in the infant [37, 61, 64].

### Ergots and ergots alkaloids

Ergots and ergots alkaloids are contraindicated in pregnancy due to a known uterotonic and vasoconstrictive effect as well as a range of serious adverse effects on the fetus like fetal distress and birth defects [18, 37, 50, 57, 59, 61, 62, 66]. Other possible teratogenic effects include intestinal atresia and poor cerebral development [3].

They should be avoided in nursing women. Beside systemic side effects in the infant like vomiting, diarrhea and convulsions, prolactine can be decreased by these drugs, reducing the milk production [37, 59, 61, 64].

#### Antiemetics

Antiemetics are believed to be mostly safe during pregnancy [21]. However, only little data are available.

Metoclopramide is commonly used during pregnancy without significant fetal side effects [50, 54, 57, 59, 61, 62]. Chlorpromazine and prochlorperazine could have an increased risk for neonatal extrapyramidal or withdrawal symptoms if taken during third trimester [37], domperidon might lead to long QT syndrome [82], and under diphenhydramin, sedation and apnea after delivery are possible [64].

Doxylamine, histamine H1 receptor antagonists, pyridoxine, dicycloverine and phenothiazines are other options without noted adverse pregnancy outcomes [54]. Recently some concerns arised on the use of ondansetron during pregnancy. There seems to be conflicting evidence about a possible teratogenic effect as well as the potential to cause a serotonin syndrome and QT prolongation [37].

A potential toxicity of metaclopramide, chlorpromazine and prochlorperazine for the infant is suspected when used in nursing mothers [57, 59, 64]. Antiemetics could lead to sedation or irritability, but also apnea and extrapyramidal symptoms are possible [37].

Based on the above mentioned informations paracetamol 500 mg alone or in combination with aspirin 100 mg, metoclopramide 10 mg, or tramadol 50 mg are recommended as first choice symptomatic treatment of a moderate-to-severe primary headache during pregrancy. Sumatriptan may be the second choice symptomatic treatment for migraine in pregnant women.

### Preventative treatment

#### Antihypertensive drugs

Β-blockers (metoprolol and propranolol) are the first line option as migraine prophylaxis in pregnant and breastfeeding women. [37, 54, 59–61]. Potential fetal side effects like intrauterine growth retardation, preterm birth and respiratory distress are described in some studies [37, 60]. If possible a prelabour tapper off should be achieved as the use of β-blockers in the third trimester can induce neonatal pharmacological effects like bradycardia, hypotension and hypoglycaemia. Newborns exposed in the last trimester should be closely monitored [37, 61].

Β-blockers are excreted in breast milk in very low doses and infant plasma concentrations are negligible. When nursing, metoprolol is preferred over propranolol. Possible side effects include drowsiness, neonatal hypoglycemia, hypotension, weakness and bradycardia [37]. Caution has to be paid in infants with astma [64].

Antihypertensive drugs which interfere with the renine-angiotensine system, like the ACE inhibitor lisinopril or the angiotensin-receptor blocker candesartan, are considered contraindicated at any stage of pregnancy since their use involves a significant fetal risk [37, 61]. Candesartan is probably compatible with breastfeeding with special attention for kidney development [64]. Lisinopril seems probably compatible as well, but there is no specific breastfeeding data available [64].

When prophylactic treatment is needed in a pregnant or breastfeeding CH patient, verapamil in the lowest effective dose remains the first choice [57, 64, 79, 80].

#### Antidepressants

The tricyclic antidepressants (TCA) are considered the safest second-line option when β-blockers are contraindicated or ineffective. Amitriptyline is preferred. Some studies suggest a possible teratogenic effect of TCA (e.g., cardiovascular or limb abnormalities), but a clear causal relationship can not be proven [18, 37, 59, 61, 62]. If used late in pregnancy, all antidepressants might lead to withdrawal symptoms [82]. Amitriptyline and nortriptyline are relatively safe during breastfeeding. In mothers treated with amitriptylin, infants are exposed to about 1–2% of maternal dose and no accumulation is supposed [60] and this does not seem to have a negative impact on the child [59–61, 64]. However, drowsiness and anticholinergic symptoms like dry mouth or constipation might occur [37].

The seretonin norepinephrine reuptake inhibitor (SNRI) venlafaxine should be avoided during pregnancy. There is no clear indication of a possible teratogenic or abortifacient effect of duloxetine. No adverse pediatric effects have been reported in the little data on nursing infants of mothers using the snris duloxetine and venlafaxine [50, 60, 62, 64].

#### Antiepileptic drugs

Valproate is contraindicated during pregnancy because of devastating fetal side-effects like neural tube defects and other major malformations such as cleft palate, cardiac or urinary tract defects [37, 54, 61, 83]. Valproate transfers to breast milk in very low doses and is unlikely to affect the child seriously [37]. Although valproate seems safe when breastfeeding, it should be avoided in women of childbearing age because of its teratogenic effect. When nursing on valproate can not be avoided, monitoring for liver and platelet function in the child is advised [59, 61, 64].

The use of topiramate in pregnancy is associated with an increased risk of cleft lip/palate and low birth weight, especially when taken during first trimester [81]. The possible benefits as a migraine prevention do not seem to outweigh the risks. Therefore topiramate should be avoided in this context [37, 54, 60, 62, 84]. Topiramate reaches infant plasma level up to 25% of maternal levels and newborns should be monitored for sedation, irritability, poor suckling, weight loss and diarrhea. No other significant side effects have been reported [61, 64, 84].

Data on prenatal exposure to gabapentin are limited. A link with osteological deformities is suspected. Therefore, its use is not recommended during pregnancy [54, 60]. Gabapentin seems compatible with breastfeeding. No special concerns were reported to date [64].

Lamotrigine has a good safety profile compared with other antiepileptic drugs, therefore, it is the preferred option for women of child-bearing age. A recent meta-analyses found that rates of miscarriages, stillbirths, preterm deliveries, and small for gestational age neonates are not increased after in-utero exposure to lamotrigine compared to the general population [85]. Similarly, in-utero exposure to lamotrigine does not seem associated with increased rates of inborn defects and long-term neurodevelopmental damage [85].

The treatment with lamotrigine during breastfeeding is safe and no serious adverse effects or cognitive and development alterations have been reported [86].

#### Dietary supplements

Magnesium (up to 350 mg/die) can be used during pregnancy [81]. However, transient neurological symptoms in newborns and hypotonia have been reported [82]. If magnesium is administered intravenously over a long time, bone abnormalities are possible [37]. Due to these findings chronic use of magnesium during pregnancy seems more controversial now than before [37, 62].

Coenzyme Q10 seems a reasonable option for preventative treatment while pregnant. Up to date there are no severe adverse events reported [37, 58].

Feverfew (*Tanacetum parthenium*), butterbur (*Petasites hybridus*) and high dosed riboflavine are not recommended during pregnancy [37, 61].

Both magnesium and riboflavine are compatible with breastfeeding. About the safety of coenzyme Q10, feverfew and butterbur in lactation no clear information is available [61, 64].

#### Flunarizine

Calcium channel blockers should be avoided in pregnancy and breastfeeding, since there are not enough safety data [37, 54, 61].

#### Lithium

Lithium in CH should generally not be used in pregnancy because of a known teratogenic effect. It is associated with congenital cardiac malformations and cardiac arrhythmias, anomalies of the central nervous sytem and endocrine system, polyhydramnios and stillbirth. Use of lithium can be considered in pregnant severe CH patients when verapamil is ineffective, if the benefit to the mother clearly exceeds the possible risk to the fetus [57, 62, 79, 80].

Lithium reaches a high relative infant dose of 50%. Mainly the kindney in newborns seems sensitive to lithium and renal toxicity is described [57].

Prescription of lithium in lactating women is controversial but as in pregnant women, in cases of severe uncontrolled CH it can be considered [57, 79].

#### Botulinum toxin type a

Botulinum toxin type A is probably safe during pregnancy due to its local mechanisms of action. However, only very few data are available and mainly for its use as cosmetic treatment [61].

There are no reports for botulinum toxin type A during breast-feeding but a transfer to breast milk is not probable due to its high molecular weight [61].

As no well-controlled data is available for his indication for now it should only be reserved for severe treatment-refractory chronic migraine patients [37, 61, 87].

#### Nerve blocks

Periferal nerve blocks are considered safe in pregnancy and breastfeeding. Due to their periferal localization, the systemic effect is considerably smaller than in oral medication. The preferred agent to inject is lidocaine. Alternatives are bupivacaine or betamethasone. Bupivacaine may be associated with fetal cardiotoxicity [37, 57, 62, 88, 89].

#### Melatonin

There is no clear evidence of harmfull adverse events when using melatonin during pregnancy. However it is suggested that administration of exogenous melatonin during pregnancy can interfere with the development of the postnatal circadian rhythm [79].

Melatonin is a natural substance of breastmilk and is excreted in a circadian cycle. Hypotheticaly the use of exogenous melatonin can be thought to have a negative influence on postnatal sleep patterns and other hormonal cycles. There is no relevant data available to support this hypothesis [79]. Melatonin in low doses seems compatible with breastfeeding [64].

## Headache-related complications during pregnancy

Headache during pregnancy requires particular attention because it can be a symptom of secondary conditions, including CVT, hypertensive disorders, stroke and pituitary apoplexy [50]. At the same time, preexisting primary headache conditions can influence the course of pregnancy and delivery, and lead to a higher risk of complications [18].

### Pregnancy complication in migraine patients

Most previous literature focused on the effects of migraine on pregnancy, while other headache disorders were often neglected. In general, a preexisting migraine does not represent a risk factor for negative pregnancy outcome and no increase rate of fetal malformations could be detected in pregnant women suffering from migraine [3, 41]. However, migraine can be considered an important risk factor for hypertensive and vascular diseases during pregnancy [90].

The largest study to investigate the relationship between migraine and pregnancy complications was conducted by Bushnell et al. In form of a retrospective, population based case-control study on 18,345,538 pregnancies in the United States between 2000 and 2003 [91]. 33,956 (0.2%) of the examined pregnant women had a migraine diagnosis. The authors detected a strong correlation between migraine and vascular diseases. In particular, the risk for stroke was 15-fold higher, with odds ratios of 30.7 for ischemic and 9.1 for hemorrhagic stroke. Other vascular conditions at elevated risk were myocardial infarction and other heart diseases (OR 2.1), thromboembolic conditions (OR 3.2), hypertension (OR 8.6), pregnancy-hypertension and preeclampsia (OR 2.3) [91].

Banhidy et al. Conducted a similar study analyzing retrospectively data from the Hungarian Case-Control System of Congenital Abnormalities between 1980 and 1996 [92]. They collected data from 38,151 infants, 713 (1.9%) of them were born from mothers with a migraine diagnosis. The risk of congenital malformations was not increased but migraine was associated with a 1.4-fold higher risk of preeclampsia [92]. Also Chen et al. Detected in a retrospective study on 4911 Taiwanese women with migraine an elevated risk for preeclampsia (OR 1.3), when compared to 24,555 women without migraine. Moreover, they detected an elevated risk for preterm birth (OR 1.24) and low birth weight (OR 1.2) [93].

Comparable results regarding elevated risk for preeclampsia were collected by Simbar et al. In a retrospective study on 180 Iranian pregnant women; those with a history of migraine had a 2.7-fold higher risk for developing preeclampsia [94].

In one prospective study, Facchinetti et al. Examined the data of 702 pregnant women who were normotensive before pregnancy; the 38.5% of them reporting migraine headache had a 2.8-fold higher risk of developing hypertensive disorders during pregnancy [95]. This risk was particularly elevated for women with active migraine during pregnancy and remained significant even after adjusting for other common risk factors for hypertension such as age, smoking and positive family history. Women with migraine were also at a 1.9-fold higher risk for giving birth to low birth weight infants [95].

Similar results were reported in a more recent, smaller study by Grossman et al. [96]. They analyzed retrospectively the data of 86 pregnant women with migraine, who gave birth between 2009 and 2014. Their cohort consisted mostly of African-American and Hispanic women. In comparison with national averages, patients experiencing severe migraine attacks during pregnancy had a higher rate of complications during pregnancy and delivery, including preeclampsia (21.3% vs. 4%), preterm delivery (28.0% vs. 11.4%) and low birth weight (18.7% vs. 8%) [96]. Moreover, if migraine patients develop preeclampsia, they have also an increased risk for cerebral palsy and perinatal death [97].

The relationship between migraine and other vascular conditions remains unclear and is most probable related to overlapping pathophysiological mechanisms [21]. In the mentioned studies, the authors discuss possible common etiological backgrounds, including platelet hyperaggregation, decreased prostacyclin production, altered vasoreactivity and endothelial dysfunction [3, 98]. Supposedly, women with migraine have poor vascular compensation mechanisms in stress situations like pregnancy, leading in return to a higher incidence of vascular complications [91].

The risk of developing hypertensive disorders appears particularly high if other comorbidities are present. Czerwinski et al. Detected a 2.5-fold higher risk of developing pregnancy-induced hypertension in patients with migraine and additionally asthma [99]. Also comorbid mood-disorders can increase the risk of preterm birth and hypertensive disorders in pregnant migraineurs [100].

Furthermore, migraine is associated with a higher risk of nausea and hyperemesis gravidarum [92, 101]. Pregnant migraineurs complain significantly more often about short sleep duration, excessive daytime sleepiness, vital exhaustion and elevated perceived stress [102, 103].

Patients with migraine also have an elevated risk for depression during pregnancy and increased rates of anxiety and stress, especially in cases of migraine with aura [100, 103]. Some authors suggest that migraine and depression may share a common pathophysiology with dysfunction in the serotonergic and dopaminergic system [100].

### Pregnancy complications in patients with other headache conditions

Only few studies included pregnant patients with other primary headache conditions than migraine. Maggioni et al. Conducted a retrospective study on 430 women after delivery: 126 (29.3%) suffered from primary headache disorders, among them 81 had MO, 12 MA and 22 TTH [5]. They detected no differences in APGAR scores (a method to quickly summarize the health of newborn children) and malformations between women with and without primary headaches, regardless of the headache subtype [5].

Sanchez et al. Observed that history of headache correlated significantly with placental abruption, i.e. The separation of the placenta from uterus before delivery [104]. The odds ratio for MA was 1.59, for MO was 2.11 and for TTH was 1.61 [104].

Finally, Marozio et al. Conducted a prospective study on 376 pregnant women with headache compared to 326 pregnant women without headache [105]. Among women with headache, 264 had migraine with or without aura and 103 a TTH. Preterm deliveries occurred significantly more often within the headache group and headache subtypes did not differ regarding pregnancy complications [105].

In conclusion, migraine is a risk factor for pregnancy complications, particularly vascular events [39, 97]. The risk of gestational hypertension and preeclampsia is increased in pregnant migraineurs and active migraine during pregnancy is associated with increased risk for stroke, cardiac diseases and thromboembolic events [39]. Therefore, migraineurs should be considered at higher risk for complications during pregnancy and monitored closely [39].

Data regarding other primary headache conditions are scarce. Although some authors suggested a higher risk for pregnancy complications in all headache patients regardless of the subtype, further research is needed to validate these results and examine possible common etiological factors [105].

## Diagnostics of headache in pregnancy

Headache during pregnancy can be both primary and secondary, and in the last case can be a symptom of a life-threatening condition. During pregnancy, migraine and TTH are most common, however, there may occur conditions that resemble primary headaches but are symptoms of disorders first appearing during pregnancy. Early diagnostics of the disease manifested by headache is important for mother and fetus life.

For a differential diagnostics of headaches is important to collect the anamnesis: it is necessary to investigate family aptitude to headache, the age of its debut, whether it is a new or emerging condition; it is also important get a detailed description of its episode and its accompanying symptoms. The presence of headache before pregnancy is an important predictor of its development during or after pregnancy. It is also important to find out possible associated diseases that could trigger or worsen the course of headache. In addition, it is mandatory to consider administered medications. If there is a suspicion of the symptomatic character of the headache, it is necessary to carry out a neuroimaging, lumbar puncture and other methods [38].

### Primary headaches during pregnancy

When MA occurs for the first time during pregnancy, it is necessary to conduct brain MRI to prevent ischemic stroke. Generally, migraine does not affect the outcome of pregnancy, but it is more likely to experience premature birth and preeclampsia [25, 106].

In addition to migraine during pregnancy, TTH is also quite common. The nature of pain, its localization, duration, as well as the conditions under which pain occurs, worsens and weakens play role in its diagnostics. In case of prolonged headache not improved by analgesics, it is advisable to perform ophthalmoscopy and brain MRI to exclude volume formations and intracranial hypertension.

Cluster headache is a relatively rare type of headache. Its extensiveness is approximately 0.06% of the population, with a total ratio of men to women of approximately 2.5:1 [57]. To exclude the secondary nature of the headache, it is also advisable to perform brain MRI.

### Secondary headaches during pregnancy

It is especially important to identify “red flags” suggesting that headache is a symptom of a serious disease (Table 2). In these cases, electroencephalography, ophthalmoscopy, ultrasound of the vessels of the head and neck, brain MRI and MR angiography may be needed [38]. In some cases, it is possible to perform multi-slice computed tomography (MSCT). The risk for the fetus in this case is minimal and the contraindication is the mother allergy to the contrast agent [107].

Clinically, the most significant causes of the secondary headache in pregnant women are: stroke, subarachnoid hemorrhage, cerebral venous thrombosis, arterial dissection, pituitary tumor, choriocarcinoma, eclampsia, preeclampsia, posterior reversible encephalopathy syndrome, idiopathic intracranial hypertension, and reversible cerebral vasoconstriction syndrome.

#### Stroke (acute cerebrovascular accident)

To identify the etiology and make a diagnosis of ACA in pregnant women and puerperas, one or more of the following methods can be required: MRI and angiography, computed tomography (CT), MSCT, ophthalmoscopy electrocardiography and echocardiography, daily monitoring of arterial pressure and electrocardiograms, ultrasound examination of extra- and intracranial vessels with duplex scanning, and cerebral angiography [108].

#### Subarachnoid hemorrhage

Subarachnoid hemorrhage is caused by rupture of aneurysm or vascular malformations (arteriovenous malformation, cavernous or venous hemangioma). Intracerebral hemorrhages in pregnant women are rare. The risk of SAH is especially high in the first few days after birth. Diagnosis is confirmed by non-contrast-enhanced CT scan, which has a sensitivity of 98% in the first 12 h after onset [42]. If CT results are non-diagnostic, a lumbar puncture is essential. MRI is not indicated as an initial diagnostic test for SAH but may be useful when the CT is normal and the CSF abnormal [42]. Also, cerebral angiography can be performed, which allows determining the number of aneurysms and arteriovenous malformations, as well as their localization [108].

#### Cerebral venous thrombosis

CVT is a serious secondary headache disorder that can occur with pregnancy, usually during the 3rd trimester and postpartum period. Given the absence of specific characteristics, any recent persisting headache should raise suspicion, particularly in the presence of risk factors for CVT, such as hypertension, prothrombotic conditions, cesarean delivery, advanced maternal age, infections, and excessive vomiting. The rate of death with CVT is 2–10%, but less when associated with pregnancy [109]. Diagnosis is based on neuroimaging: MRI plus MRA, or CT scan plus CT angiography, and intra-arterial angiography in doubtful cases.

#### Arterial dissection

Headache associated to arterial dissections has no constant specific pattern and it can sometimes be very misleading, mimicking other headaches such as migraine, CH or primary thunderclap headache [42]. A painful Horner’s syndrome, painful tinnitus of sudden onset or painful xiith nerve palsy are highly suggestive of carotid artery dissection. Cervical artery dissection may be associated with intracranial artery dissection, which is a potential cause of or intracranial haemorrhages (subarachnoid or intracerebral).

Headache attributed to cervical arterial dissection usually precedes the onset of ischaemic signs, and therefore requires early diagnosis and treatment. Diagnosis is based on cervical MRI, Duplex scanning, MRA and/or CTA and, in doubtful cases, conventional angiography [42]. Several of these investigations are usually needed as any of them can be normal.

#### Pituitary tumor

Pituitary tumors account for 10% to 22% of all neoplasms of the brain. One of the most common pituitary tumors is prolactinoma. In most cases, the pathology proceeds without any apparent symptoms. Visual disturbance and headaches occur only when the size of the tumor increases and is more than one centimeter. During pregnancy it is occurs rarely.

Microadenoma and pregnancy are poorly compatible with each other. In many cases, spontaneous abortion occurs in the first trimester. The most severe complication of the increase in size of a pituitary adenoma is apoplexy, resulting from hemorrhage or infarction of the tumor, which is usually accompanied by acute headache, visual impairment and pituitary dysfunction [110].

A pregnant woman with prolactinoma must be examined every 3 months and it is necessary to find out the presence of symptoms of tumor growth: headache, visual field disturbances, and abnormalities at the ophthalmoscopy [111]. MRI is performed only with the appearance of clinical symptoms indicating a tumor growth [112].

#### Choriocarcinoma

In Europe and North America, choriocarcinoma affects approximately 1 in 40,000 pregnancies [113]. A delay in diagnosis may lead to metastatic organ damage. When metastasizing in the central nervous system, there is headache, intense dizziness, darkening in the eyes, or other symptoms of volume formation in the brain. For diagnosis, brain MRI or CT scan can be sufficient and, if they result negative, a lumbar puncture can be performed. The determination of the concentration of chorionic gonadotropin (HCG) in the cerebrospinal fluid (CSF) and blood allows detecting initial metastases. HCG poorly penetrates the blood-brain barrier. A ratio of HCG concentration in the blood and in CSF less than 40:1 indicates the involvement of the CNS [114].

#### Headache associated with preeclampsia and eclampsia

Headaches are noted in 2/3 of all patients with preeclampsia or eclampsia [115, 116]. Preeclampsia is a preconvulsive condition characterized by a significant rise in blood pressure, a high protein concentration in the urine, and significant edemas. It occurs after the 20th week of pregnancy or in the postpartum period. Eclampsia is a convulsive seizure, which is preceded by a headache and a flash of light feeling. This state is either allowed or goes into a coma. Risk factors for the development of preeclampsia and eclampsia are overweight, hypertension, age over 40, diabetes, kidney disease, and multiple pregnancies. The occurring of preeclampsia increases the risk of hemorrhagic stroke developing during pregnancy and childbirth [117]. In addition, it is important to know that eclampsia is the most common cause of death of pregnant women [87, 118]. In women with eclampsia, seizures are preceded by headache similar to migraine, with pulsating character, different localization, accompanied by nausea or vomiting, photophobia and phonophobia [92]. The delayed postpartum eclampsia may occur within 1 week after childbirth, and its most common symptom is headache [119]. Each pregnant woman after 20 weeks of pregnancy suffering from a difficult-to-maintain headache should be examined for preeclampsia [120–124]. Diagnostic criteria for headache associated with preeclampsia and eclampsia are presented in the ICHD-3beta [42].

#### Posterior reversible encephalopathy syndrome

The clinical symptoms are usually non-specific and the differential diagnosis of PRES in pregnancy and puerperium may be challenging. The presentation can be mistaken for other conditions such as eclampsia, ischaemic and haemorrhagic stroke, CVT, RCVS, cerebral artery dissection, metabolic and demyelinating disorders, vasculitis and encephalitis [125]. CT imaging in PRES show lesions in only about 50% [126]. MRI represents the gold standard for this condition and leads to the correct diagnosis in most cases and may, therefore, forestall further investigations. Typical findings are bilateral and symmetrical white matter vasogenic oedema in the parieto-occipital regions; however, lesions can occur in both white and grey matter and can involve the frontal and temporal lobes, basal ganglia, brain stem and cerebellum [127].

#### Primary and secondary intracranial hypertension

The symptoms of idiopathic intracranial hypertension (IIH) are daily progressive non-pulsating headaches, which increase with the change in the body position and also with the Valsalva probe, with a transient feeling of darkening in the eyes and pulsating noise in the ears [121, 128, 129]. Most often IIH occurs in women of childbearing who are overweight. For the diagnosis is necessary to determine the absence of the brain substance defects and signs of thrombosis of the brain sinuses, therefore it is necessary to carry out brain MRI and MR angiography. In addition, it is necessary to define the absence of high pressure of CSF and changes in its composition. An important diagnostic sign is edema of the optic nerve disk and progressive diplopia, which in the absence of adequate therapy, can be irreversible [121]. However, in one out ten cases otpic disk edema may be absent in IIH, since it takes weeks or months to develop [130].

The causes of secondary intracranial hypertension (SIH) may be different, including volume intracranial formations. More than the half of cases depends on venous sinuses thrombosis. The most frequent variant are thrombosis of cortical veins, causing headaches together with focal epileptic attacks, and thrombosis of the veins of the dura mater, resulting in a series of headaches, focal epileptic seizures and focal neurological deficiency. Cerebral vein thrombosis can occur during any gestation age, but more often in the postpartum period. MRI and MSCT venography are the most informative methods for detecting thrombosis of intracranial veins and sinuses [131].

#### Reversible cerebral vasoconstriction syndrome

Reversible cerebral vasoconstriction syndrome was considered a very rare disease in pregnant women, but over the time this condition became diagnosed more often, as postpartum angiography became possible to conduct [123]. The main symptom is “thunder-like headache” at the beginning of the disease with angiographic signs of vasoconstriction. The principal risk factor is a high concentration of vasoconstrictor substances in the body of a pregnant woman. This syndrome can also develop in the postpartum period due to the use of ergometrine maleate in postpartum hemorrhage [124]. The diagnostic criteria for headaches associated with reversible cerebral vasoconstriction syndrome are presented in ICHD-3beta [42].

#### Postpartum headache

In the postpartum period, the frequency of headache increases, mostly depending on the return of migraine, but also as a result of epidural anesthesia [120]. Headache that appeared after epidural anesthesia is quite typical. It is caused by a decrease in CSF pressure, occurs unexpectedly 1 to 7 days after puncture and has a positional character. Differential diagnosis of secondary headache in the postpartum period is carried out for postpartum eclampsia and subdural hematoma [121], angiopathy [132, 133], meningitis, cerebral thrombosis of veins and sinuses [120, 134], stroke, pituitary tumor, and chorioocarcinoma.

## Conclusions

Headache is a common complaint in the general population, particularly in females. Therefore, it is not surprising that it is a frequent presentation in pregnant women. Primary headaches, such as migraine and tension headache, account for most headaches in pregnancy. Most women notice their headache either go away or greatly improve in the second and third trimesters of pregnancy, possibly due to a reduction in reproductive hormonal fluctuation. However, around 10% experience a worsening of symptoms and after delivery, most women quickly return to their pre-pregnancy migraine pattern.

Pregnancy creates alterations in maternal physiology that increase the risk of several dangerous secondary headache disorders, especially those associated with vascular endothelial dysfunction and hypertensive disorders of pregnancy. It is fundamental to consider secondary causes in the differential diagnosis of headache, which may require urgent investigation. Pre-eclampsia, eclampsia, CVT, certain types of ischemic and hemorrhagic stroke, SAH, pituitary apoplexy, RCVS, PRES, and thunderclap headache show an overlapping clinical presentations and need to be treated emergently. One or more between electroencephalography, ultrasound of the vessels of the head and neck, brain MRI and MR angiography with contrast, brain CT, ophthalmoscopy and lumbar puncture will distinguish primary and secondary headaches.

Pregnancy and lactation can complicate treatment options for women with migraine because of the risk of certain medications to the fetus and because medications are passed on in a mother’s milk to varying degrees.

Paracetamol use in pregnancy is safe and ibuprofen can be prescribed for short-term use in the first and second trimesters. There are increasing safety data on triptans to treat migraine in pregnancy and, if other treatments have failed, sumatriptan may be used to treat acute migraine attacks also while nursing.

Options in prescription preventive medications are limited and it may be best to consider the safest interventions, which are lifestyle changes and behavioural treatment for stress management. When preventive pharmaceutical treatment is needed for migraine metoprolol and propranolol are the first choice followed by amitriptyline. Little data are available for Botulinum toxin type A use.
